# A novel classification system for anterior femoral notching in total knee arthroplasty

**DOI:** 10.1186/s42836-026-00424-4

**Published:** 2026-09-03

**Authors:** Alexander Antoniadis, Jaad Mahlouly, Thibaut Royon, Julien Wegrzyn

**Affiliations:** Department of Orthopedic Surgery and Trauma, Lausanne University Hospital and University of Lausanne, Lausanne, CH-1011 Switzerland

**Keywords:** Total knee arthroplasty, Femoral notching, Trochlear GEOMETRY, Implant fit, Biomechanics, Intraoperative assessment

## Abstract

**Background:**

Femoral notching in Total Knee Arthroplasty (TKA) is traditionally associated with an increased risk of periprosthetic fracture in cases of excessive anterior resection. Beyond fracture risk, notching can influence implant fit, patellofemoral and tibiofemoral balance, joint stability, and overall biomechanics. Despite its clinical importance, a standardized system for describing femoral notching is lacking. We propose a novel Femoral Notch Classification, based on resection depths and trochlear geometry, intended as a common language applicable both intraoperatively and on lateral radiographs, independent of alignment philosophy.

**Methods:**

The classification was developed through clinical observation and biomechanical rationale, focusing on preserving anterior cortical integrity while permitting a controlled notch when required. Definitions relate resection depths to implant thickness, distal femoral anatomy, and trochlear geometry, with attention to balancing anterior and posterior gaps. Perpendicularity of the anterior cut relative to the cortex can be evaluated and adapted according to implant design and alignment technique. Four descriptive categories were established, providing a structured framework for assessment.

**Results:**

(1) Type 0—No Notch: Complete preservation of the anterior cortex with no visible step-off or indentation. Anterior resection may match implant thickness precisely; if not, anterior overstuffing or posterior over-resection should be controlled. (2) Type 1—On the Line: Anterior resection is flush with the femoral cortex, creating a subtle notch that remains biomechanically stable without compromising the cortex. (3) Type 2—Controlled Notch: A deeper anterior resection (ideally, < 3 mm) beyond the cortical line in the presence of specific trochlear geometry or distal femoral anatomy. This configuration is acceptable when the radiographic pattern is consistent with trochlear accommodation. Posterior gaps must still be carefully controlled to avoid overstuffing. (4) Type 3—Critical Notch: Excessive anterior resection (> 3 mm) characterized by significant cortical interruption and the absence of a radiographically identifiable anatomical correlate. This configuration may compromise distal femoral strength and is associated with increased risk of periprosthetic fracture when not justified by trochlear geometry. Surgeons should exercise caution and verify proper implant alignment. Inter-observer reliability was substantial (Fleiss’ κ = 0.80; quadratic weighted κ = 0.87) and intra-observer reliability was almost perfect (mean Cohen’s κ = 0.91). The majority of cases were classified as Type 0 and Type 1.

**Conclusion:**

The Femoral Notch Classification provides a practical, reproducible framework for assessing femoral notching in TKA, suitable for intraoperative guidance and radiographic analysis. It may help surgeons avoid over- or under-resection while allowing a controlled notch when required. Further clinical validation is needed to assess its impact on long-term patient outcomes.

## Introduction

Anterior femoral notching (FN) during total knee arthroplasty (TKA) has historically been viewed primarily as a risk factor for postoperative supracondylar periprosthetic fracture, with large series reporting fracture prevalence and clinical outcomes after notching [1]. Biomechanical studies have demonstrated that notches deeper than 3 mm can reduce distal femoral strength by approximately 18 to 31%, particularly when cortical integrity is compromised or bone quality is reduced [2–4]. More recent clinical data, however, suggest that not all notches correlate with higher fracture incidence, indicating that risk is modulated by notch depth, cortical involvement, and patient factors rather than by the mere presence of a notch [5, 6].

Beyond fracture risk, femoral notching has implications for implant fit, patellofemoral balance, joint stability, and overall biomechanics. Computational finite element analyses and gait simulations demonstrate that notch depth alters distal femoral stress distribution during the gait cycle, underscoring the need to coordinate anterior and posterior resections to avoid over- or under-resection of the compartments [7]. Techniques prioritizing restoration of distal femoral anatomy with minimal compromise—such as anatomic or hybrid referencing—aim to balance anterior and posterior gaps, reduce unintended cortical violation, and optimize patellofemoral mechanics [8].

These decisions are best grounded in standardized radiographic documentation. The modern Knee Society methodology has formalized recording of alignment and bone status on routine anteroposterior and lateral radiographs, while calls for uniform assessments in revision arthroplasty highlight the value of shared criteria across centers and implant designs [9, 10]. In parallel, radiological taxonomies of distal femoral morphology demonstrate that reliable, image-based classification is feasible for establishing common terminology [11, 12].

Despite its importance, no widely adopted, radiograph-based classification is dedicated to anterior FN in TKA. The Tayside scheme remains the most recognized for postoperative radiographic grading by extent of cortical and cancellous involvement, but it offers limited intraoperative guidance, and many reports still describe notching qualitatively (present/absent; mild/moderate/severe) without a standardized framework [6]. More broadly, preoperative planning principles and bone-defect classifications underscore why a common radiographic language matters: uniform description improves anticipation of technical challenges, supports reproducible restoration of femoro-tibial alignment and patello-femoral congruence, and facilitates comparison of outcomes across studies and implants [13, 14].

We propose a descriptive, radiograph-based Femoral Notch Classification intended as a common language for routine reporting after total knee arthroplasty. The present work defines the taxonomy and its radiographic criteria.

## Methods

### Development of the classification

The FN Classification was developed through biomechanical understanding and extensive clinical observation, with the primary goal of preserving maximal anterior cortical integrity while permitting a radiographic pattern consistent with a controlled notch when anatomically indicated. These biomechanical observations are derived from previously published literature and are not newly evaluated in the present study, which focuses on classification development and preliminary reliability testing. Common indications for a controlled notch include variations in distal femoral anatomy—such as pronounced anterior curvature, a shallow or dysplastic trochlea, or mismatch between native trochlear geometry and implant design—that would otherwise lead to anterior overstuffing or compromised patellofemoral tracking if strictly avoided.

The concept arose from the consistent observation that postoperative lateral radiographs demonstrate reproducible patterns of anterior femoral morphology after TKA. These patterns range from extensive anterior cortical loss to a smooth anterior cortical profile that appears compatible with the patient's native anterior femoral anatomy while preserving the posterior cortex. These reproducible radiographic appearances formed the basis for the proposed classification. The classification was therefore designed as a descriptive, radiograph-based framework intended to be applicable both intraoperatively (independent of implant design or alignment philosophy) and on postoperative lateral radiographs. Differentiation between Type 2 and Type 3 was based on notch depth measured from the anterior cortical line on true lateral radiographs, preservation of cortical continuity, and comparison with preoperative radiographs to assess underlying trochlear geometry. Notch depth was defined as the maximum perpendicular distance between the anterior cortical line and the anterior femoral cut. A true lateral radiograph with overlapping posterior condyles was required for reliable notch depth measurement. The 3-mm threshold was used as a practical reference point based on previous biomechanical studies.

### Radiographic assessment and classification criteria

All definitions are anchored to standard anteroposterior and lateral radiographs obtained according to the Modern Knee Society radiographic methodology for TKA [9]. On the true lateral projection, the anterior cortical line, the anterior resection plane, and the distal anterior flange of the femoral component are analyzed. The system relates the depth and configuration of anterior femoral notching (FN) to implant thickness, trochlear geometry, and the balance between anterior and posterior resections, thereby defining reproducible geometric patterns that reflect the degree of cortical preservation or violation. Classification was based primarily on qualitative radiographic patterns rather than absolute quantitative measurements. The 3-mm depth was used as a practical reference threshold; final classification also incorporated cortical continuity and comparison with preoperative radiographs.

The classification comprises four descriptive types (Table 1).

**Table 1:** The proposed anterior femoral notching classification

Type	Name	Key radiographic features	Clinical implication
Type 0	No Notch	Complete preservation of the anterior cortex; anterior resection might match implant thickness; no visible step-off or indentation	Safe configuration with complete cortical preservation; posterior over-resection should be avoided to maintain flexion–extension gap balance
Type 1	On the Line	Resection flush with native cortex; subtle contact, minimal or no measurable cortical loss	Safe, biomechanically stable configuration with full cortical preservation; represents neutral balance of implant thickness and native anatomy
Type 2	Controlled Notch	Shallow resection beyond cortical line (ideally < 3 mm); smooth with preserved continuity, consistent with underlying anatomy	Acceptable when anatomically indicated (e.g., trochlear mismatch); posterior gaps must be controlled to avoid overstuffing
Type 3	Critical Notch	Excessive anterior resection (> 3 mm) characterized by significant cortical interruption and the absence of a radiographically identifiable anatomical correlate	Potentially increased fracture risk and mechanical compromise; requires intraoperative verification of alignment and sizing

### Validation of the classification

The reliability of the proposed Femoral Notch Classification was assessed using 80 anonymized true lateral radiographs from primary total knee arthroplasties performed at our institution. All radiographs met the standards of the Modern Knee Society radiographic evaluation system. Three independent observers (one senior orthopedic surgeon and two orthopedic fellows) classified each radiograph into one of the four types (Type 0–3) according to the provided definitions and example figures. Observers were blinded to clinical information and to each other’s ratings. After an interval of four weeks, the same set of radiographs was presented in randomized order to determine intra-observer reliability. Quadratic weighted kappa was calculated as the average of the pairwise weighted kappas between the three observers. During the reliability assessment, the three observers had access to the corresponding preoperative radiographs to evaluate native trochlear geometry when classifying postoperative images.

## Results

### The Femoral Notch Classification

**Fig. 1:**
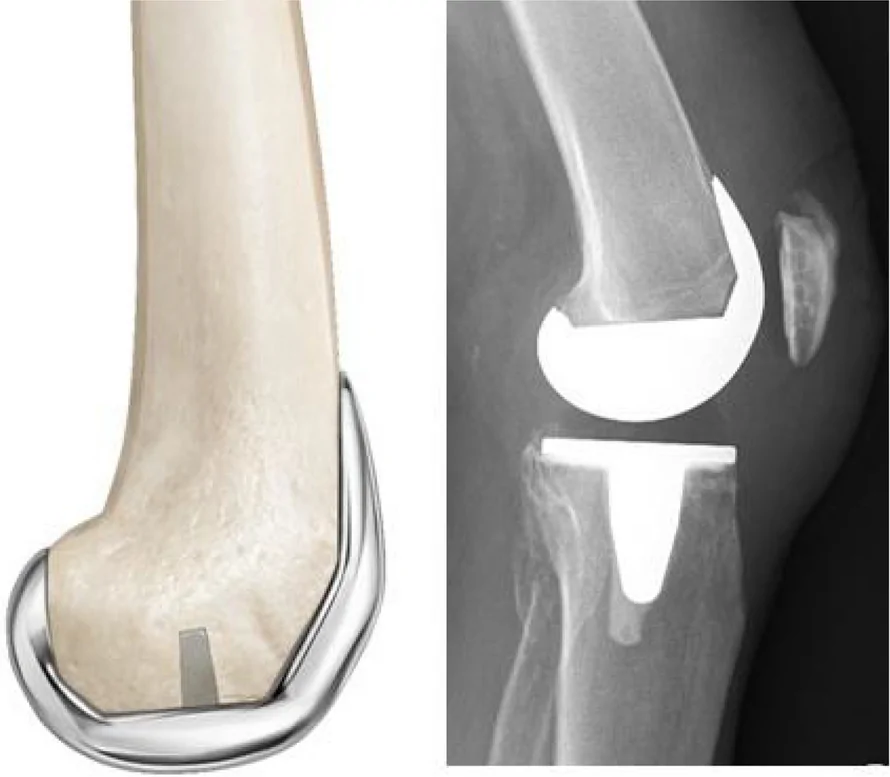
Representative example of a Type 0—No Notch pattern. Left: schematic representation showing complete preservation of the anterior femoral cortex. Right: corresponding postoperative lateral radiograph demonstrating an intact anterior cortical contour

**Fig. 2:**
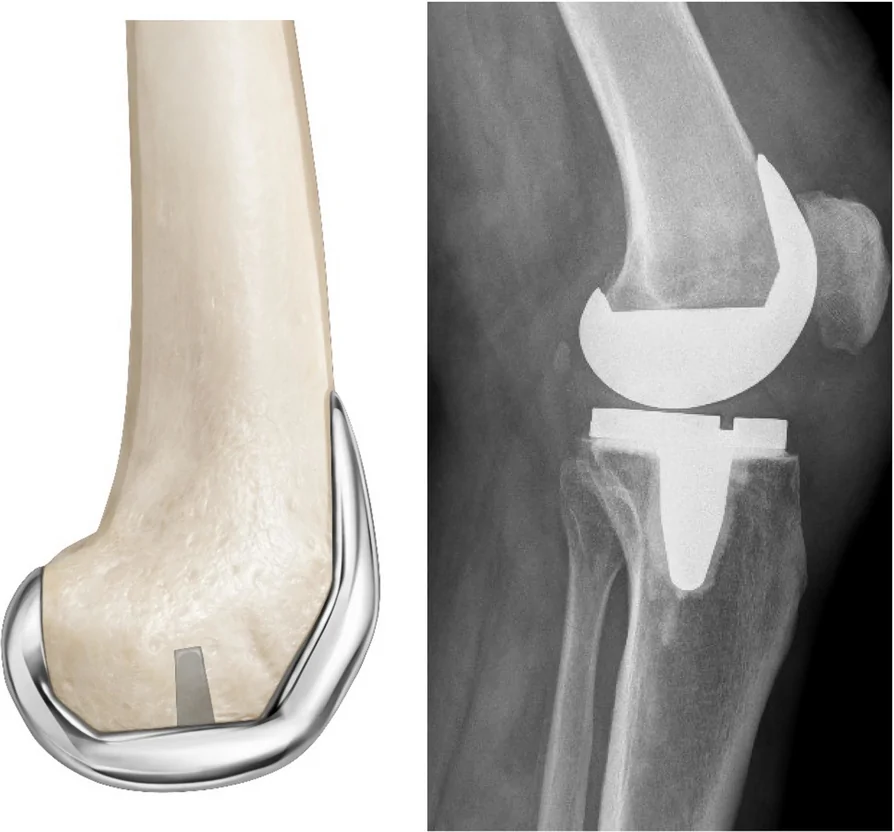
Representative example of a Type 1—On the Line pattern. Left: schematic representation showing an anterior femoral resection aligned with the native anterior cortical contour. Right: corresponding postoperative lateral radiograph demonstrating a flush cortical transition with preserved anterior cortical continuity

**Fig. 3:**
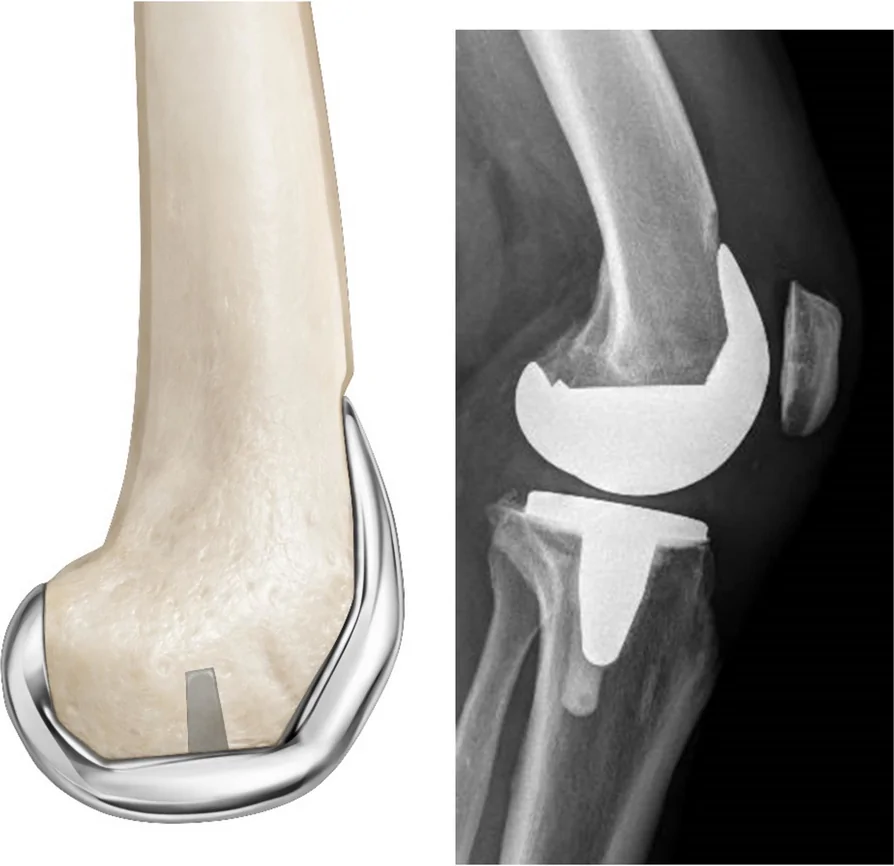
Representative example of a Type 2—Controlled Notch pattern. Left: schematic representation showing a shallow anterior cortical resection beyond the native cortical line with a smooth contour. Right: corresponding postoperative lateral radiograph

**Fig. 4:**
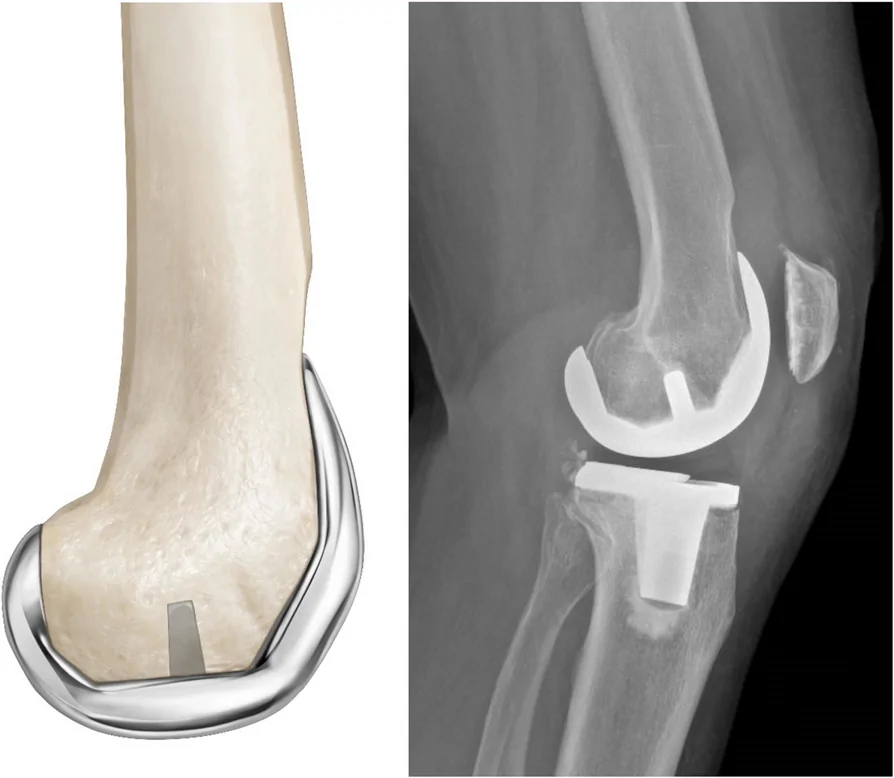
Representative example of a Type 3—Critical Notch pattern. Left: schematic representation showing excessive anterior resection (> 3 mm) with cortical interruption and no radiographically identifiable anatomical correlate. Right: corresponding postoperative lateral radiograph

The proposed classification comprises four descriptive categories (Types 0–3) based on the geometric relationship between the anterior femoral resection plane, the native cortical line, and the anterior flange of the femoral component on true lateral radiographs (Figs. 1, 2, 3 and 4).

#### Type 0—no notch

Type 0 is characterized by complete preservation of the anterior cortex with no visible step-off or indentation. The anterior resection might match precisely the femoral component thickness. The anterior contour of the implant follows the native cortical line harmoniously, although slight proximal cortical exposure may occur (e.g., when the component is placed in higher flexion). In this configuration, posterior over-resection must be carefully avoided to preserve balanced flexion–extension gaps.

#### Type 1—on the line

The anterior resection is flush with the native cortex, resulting in subtle cortical contact with minimal or no measurable cortical loss. This configuration creates a faint “on the line” appearance without true indentation and is considered biomechanically stable, fully preserving cortical integrity. It typically represents a neutral configuration achieved when the anterior cut precisely accommodates both implant thickness and native femoral curvature.

#### Type 2—controlled notch

This pattern was characterized by a deeper anterior cortical resection with a smooth contour and preserved cortical continuity, representing a radiographic appearance consistent with accommodation of trochlear geometry. The resection extends beyond the cortical line but should be deliberately smooth, shallow, and limited (ideally < 3 mm) to preserve cortical continuity. This configuration is considered acceptable when anatomically indicated. Independently, posterior under-resection must be carefully controlled to avoid overstuffing the posterior compartment.

#### Type 3—critical notch

Excessive anterior resection (> 3 mm) characterized by significant cortical interruption and the absence of a radiographically identifiable anatomical correlate. On radiographs, the cortical line is visibly interrupted, thinned, or undercut beyond the expected implant contour. This configuration compromises distal femoral mechanical strength and may increase the risk of supracondylar periprosthetic fracture. If encountered intraoperatively, as a potential application of the classification requiring future validation, surgeons may consider verifying implant alignment and sizing before final implantation.

Across all types, the perpendicularity of the anterior resection relative to the native cortex can be evaluated on lateral radiographs. This aspect allows adaptation according to implant design and may be considered as a potential extension of the classification in future studies. Overall, the classification provides a reproducible, visual framework for intraoperative decision-making and postoperative communication.

### Reliability of the Femoral Notch Classification

Inter-observer reliability among the three observers, evaluated using Fleiss’ kappa, was 0.80 (95% CI 0.73–0.86), and quadratic weighted kappa was 0.87 (95% CI 0.82–0.92), indicating substantial agreement. Intra-observer reliability, measured by Cohen’s kappa, was almost perfect, ranging from 0.87 to 0.94 (mean 0.91) (Table 2). These findings demonstrate that the proposed classification system is highly reproducible and reliable across observers with different levels of experience. In cases of doubt, particularly when distinguishing between Type 2 and Type 3, consultation of preoperative radiographs is recommended to better assess the underlying trochlear geometry and anatomic indication. The distribution of the 80 radiographs across the four types is shown in Table 3.

**Table 2:** Inter- and intra-observer reliability of the Femoral Notch Classification

Reliability type	Statistical measure	Kappa (95% CI)	Strength of agreement*
Inter-observer (3 observers)	Fleiss’ κ (unweighted)	0.80 (0.73–0.86)	Substantial
Inter-observer (3 observers)	Quadratic weighted κ	0.87 (0.82–0.92)	Almost perfect
Intra-observer (mean)	Cohen’s κ	0.91 (0.87–0.94)	Almost perfect

^*^According to Landis and Koch criteria

**Table 3:** Distribution of the 80 postoperative lateral radiographs across the Femoral Notch Classification types

Type	Number of cases	Percentage (%)
Type 0—No Notch	40	50.0
Type 1—On the Line	29	36.3
Type 2—Controlled Notch	10	12.5
Type 3—Critical Notch	1	1.3
Total	80	100

## Discussion

Femoral notching (FN) in total knee arthroplasty (TKA) has traditionally been regarded as a complication to be avoided owing to its association with supracondylar periprosthetic fracture, particularly when depth exceeds 3 mm and cortical integrity is compromised [1, 2]. However, accumulating biomechanical and clinical evidence indicates that not all FN is detrimental; in selected cases, a controlled notch may be purposeful and advantageous. A deliberate, shallow resection can prevent anterior compartment overstuffing, enhance patellofemoral tracking, and facilitate flexion–extension gap balance—especially with implants featuring prominent trochlear flanges [3, 4].

Despite its clinical relevance, no widely accepted classification distinguishes radiographic patterns of controlled from critical femoral notching (FN) or guides intraoperative decision-making. The Tayside classification, proposed by Gujarathi et al. [6], remains the most recognized system, grading postoperative notching into three levels based on depth and extent of cortical or cancellous involvement. While valuable for retrospective stratification of fracture risk—particularly for deeper notches that would correspond to Type 3 in the current system—it offers limited intraoperative utility, does not account for the subtleties of radiographic patterns (Types 0–2), and, to our knowledge, has not achieved widespread clinical or research adoption. Table 4 summarizes the main conceptual differences between the proposed classification and the Tayside system.

**Table 4:** Conceptual comparison between the proposed Femoral Notch Classification and the Tayside classification

Feature	Proposed Femoral Notch Classification	Tayside classification
Basis	Radiographic pattern, relation to implant thickness and trochlear geometry	Depth of notch and cortical/cancellous involvement
Number of categories	4 (Type 0–3)	3 grades
Intraoperative utility	High (intended to be applicable during surgery)	Limited (mainly postoperative)
Considers anatomic context	Yes (native anatomy)	No
Handling of subtle notches	Differentiates Type 0 and Type 1	No
Primary focus	Cortical preservation, gap balance, and clinical relevance	Fracture risk stratification

The proposed Femoral Notch Classification addresses these gaps by providing a framework intended to be applicable both intraoperatively and on postoperative lateral radiographs, independent of alignment philosophy. Unlike depth-focused systems, it categorizes FN according to functional characteristics—cortical integrity, pattern of trochlear accommodation, and gap balance. This approach reflects clinical reality: a shallow, symmetrical, anatomically indicated notch (Type 2) may be biomechanically stable or even beneficial, whereas an unplanned, excessive notch (Type 3) risks cortical compromise and elevated fracture potential.

Additionally, the classification accommodates variations in implant design and referencing technique. Posterior-referenced systems, for instance, may predispose to unintended notching when external rotation or sizing mismatches shift the anterior cut relative to the native cortex [15].

With the increasing adoption of robotic-assisted surgery, these technologies have demonstrably reduced the incidence of unintended FN through improved cut precision and preoperative planning. Nevertheless, a subtle, controlled notch may still be performed—even with robotic assistance—when required to accommodate challenging trochlear geometry or prevent anterior overstuffing, underscoring the continued relevance of a nuanced classification system.

Because FN type is defined by reproducible geometric relationships visible on standard lateral radiographs, the classification supports standardized reporting in research and registry data. This may facilitate more consistent outcome studies compared with current qualitative or depth-only descriptions.

In the present study, the classification achieved substantial inter-observer agreement despite including observers with different levels of experience. However, distinguishing between a controlled (Type 2) and a critical notch (Type 3) occasionally might be challenging on postoperative radiographs alone. In such cases, review of preoperative radiographs can provide valuable information regarding native trochlear geometry and help determine whether the notch was anatomically justified.

Certain limitations of the classification should be acknowledged. This reliability study was conducted at a single institution. Distinguishing between a controlled notch (Type 2) and a critical notch (Type 3) may occasionally prove challenging on postoperative radiographs alone, particularly when notch depth approaches 3 mm or cortical continuity is borderline. Given the low number of Type 2 (*n* = 10) and especially Type 3 (*n* = 1) cases, the overall inter-observer agreement is largely driven by Types 0 and 1. Consequently, reliability estimates for Types 2 and 3 should be regarded as preliminary pending larger external validation. It should be noted that the reliability assessment was performed with access to preoperative radiographs. Additionally, assessment of anatomic context based on radiographic pattern alone may be inherently subjective. Although the classification is designed to be applicable both intraoperatively and on postoperative radiographs, the reliability analysis in the present study was performed exclusively on postoperative images. Therefore, its intraoperative utility remains a proposed application that requires further validation. Finally, while the classification is designed to be implant- and technique-agnostic, subtle differences in trochlear flange design across manufacturers could influence radiographic interpretation.

Future investigations should assess external validity and correlations with clinical outcomes including supracondylar fracture rates, anterior knee pain, patellofemoral complications, and implant survival. By providing a common language for both surgical decision-making and postoperative evaluation, this classification offers a practical step toward a more refined understanding of femoral notching in contemporary TKA.

## Conclusion

The proposed Femoral Notch Classification provides a practical, reproducible framework that integrates biomechanical rationale from prior literature and radiographic pattern assessment to characterize anterior femoral notching in total knee arthroplasty. By distinguishing harmless or anatomically appropriate configurations from those potentially compromising cortical integrity, it offers surgeons a common language for intraoperative decision-making and postoperative evaluation, independent of implant design or alignment philosophy. Although promising in concept, prospective validation of its reliability and clinical impact is required. Ultimately, this classification may facilitate clearer and more standardized description and contribute to a more nuanced management of femoral notching, reducing unnecessary concern over controlled resections while maintaining vigilance against excessive cortical violation.

## Data Availability

The datasets analyzed during the current study are not publicly available due to privacy restrictions but are available from the corresponding author on reasonable request and with institutional approval.
